# Supplementary material for: Characterization and Immunomodulatory Effects of High Molecular Weight Fucoidan Fraction from the Sporophyll of Undaria pinnatifida in Cyclophosphamide-Induced Immunosuppressed Mice
Source: Mar Drugs. 2019 Jul 29;17(8):447. doi: 10.3390/md17080447 (PMC6723532; doi:10.3390/md17080447)
Supplement: Supplementary file 1 [file marinedrugs-17-00447-s001.pdf]

Table S1. Quantitative reverse transcription polymerase chain reaction (qRT-PCR) primers.

Origin	Marker	Sequence	
**mouse**	Interleukin (IL)-1β	Sense	5′-CAA CCA ACA AGT GAT ATT CTC CAT G-3′
		Antisense	5′-GAT CCA CAC TCT CCA GCT GCA-3′
	Interleukin (IL)-2	Sense	5′-CCT GAG CAG GAT GGA GAA TTA CA-3′
		Antisense	5′-TCC AGA ACA TGC CGC AGA G-3′
	Interleukin (IL)-4	Sense	5′-ACA GGA GAA GGG ACG CCA T-3′
		Antisense	5′-GAA GCC CTA CAG ACG AGC TCA-3′
	Interleukin (IL)-5	Sense	5′-AGC ACA GTG GTG AAA GAG ACC TT-3′
		Antisense	5′-TCC AAT GCA TAG CTG GTG ATT T-3′
	Interleukin (IL)-6	Sense	5′-TCC AGT TGC CTT CTT GGG AC-3′
		Antisense	5′-GTG TAA TTA AGC CTC CGA CTT G-3′
	Interleukin (IL)-10	Sense	5′-GGT TGC CAA GCC TTA TCG GA-3′
		Antisense	5′-ACC TGC TCC ACT GCC TTG CT-3′
	Interleukin (IL)-12	Sense	5′-TGG TTT GCC ATC GTT TTG CTG-3′
		Antisense	5′-ACA GGT GAG GTT CAC TGT TTC T-3′
	Tumor nec rosis factor (TNF)-α	Sense	5′-CAT CTT CTC AAA ATT CGA GTG ACA A-3′
		Antisense	5′-TGG GAG TAG ACA AGG TAC AAC CC-3′
	Interferon (IFN)-γ	Sense	5′-TCA AGT GGC ATA GAT GTG GAA GAA-3′
		Antisense	5′-TGG CTC TGC AGG ATT TTC ATG-3′
	RN18s (18S riboso mal RNA)	Sense	5′-GTA ACC CGT TGA ACC CCA TT-3′
		Antisense	5′-CCA TCC AAT CGG TAG TAG CG-3′
